# Formation of Lutein, β-Carotene and Astaxanthin in a *Coelastrella* sp. Isolate

**DOI:** 10.3390/molecules27206950

**Published:** 2022-10-17

**Authors:** Hamdy Elsayed Ahmed Ali, Fritz Vorisek, Scot E. Dowd, Stephanie Kesner, Yang Song, Dali Qian, Mark Crocker

**Affiliations:** 1 Center for Applied Energy Research, University of Kentucky, Lexington, KY 40511, USA; 2 Department of Radiation Microbiology, National Center for Radiation Research and Technology (NCRRT), Egyptian Atomic Energy Authority (EAEA), Cairo 11787, Egypt; 3 MR DNA (Molecular Research LP), 503 Clovis Road, Shallowater, TX 79363, USA; 4 Department of Chemistry, University of Kentucky, Lexington, KY 40506, USA

**Keywords:** algae, pigments, lutein, β-carotene, astaxanthin, fatty acids

## Abstract

In this study, the effect of media composition, N/P ratio and cultivation strategy on the formation of carotenoids in a *Coelastrella* sp. isolate was investigated. A two-stage process utilizing different media in the vegetative stage, with subsequent re-suspension in medium without nitrate, was employed to enhance the formation of carotenoids. The optimal growth and carotenoid content (β-carotene and lutein) in the vegetative phase were obtained by cultivation in M-8 and BG11 media. Use of a N/P ratio of 37.5 and low light intensity of 40 μmol m^−2^ s^−1^ (control conditions) led to optimal biomass production of up to 1.31 g L^−1^. Low concentrations of astaxanthin (maximum of 0.31 wt. %) were accumulated under stress conditions (nitrogen-deficient medium containing 1.5 % of NaCl and light intensity of 500 μmol m^−2^ s^−1^), while β-carotene and lutein (combined maximum of 2.12 wt. %) were produced under non-stress conditions. Lipid analysis revealed that palmitic (C16:0) and oleic (C18:1) constituted the main algal fatty acid chains (50.2 ± 2.1% of the total fatty acids), while esterifiable lipids constituted 17.2 ± 0.5% of the biomass by weight. These results suggest that *Coelastrella* sp. could also be a promising feedstock for biodiesel production.

## 1. Introduction

The combination of a growing population and the ‘westernization’ of society has begun to place stress on the ecosystem, particularly with respect to the supply of adequate energy, food, and water resources. Hence, new technologies are required to address resource demand at the water–energy–food nexus. Large-scale microalgae cultivation represents one potential approach. In this context, the relatively high photosynthetic efficiency of phototrophic microalgae and cyanobacteria, coupled with their ability to recycle waste streams such as eutrophic wastewater and carbon dioxide from fermentation and combustion processes, is of particular interest. While algae cultivation has primarily been investigated for the long-term replacement of petroleum-derived fuels, it also has the potential to generate an array of biologically derived compounds. The biodiversity of these organisms can be leveraged across a wide variety of operating conditions (salinity, temperature, pH, etc.) to facilitate the production of lipids, carbohydrates, and proteins. Furthermore, there is potential to produce high value products such as EPA/DHA and carotenoids [1,2,3].

In addition to chlorophyll as the primary photosynthetic pigment, microalgae frequently contain other pigments such as carotenoids. The primary carotenoids, such as neoxanthin, violaxanthin, lutein and β-carotene, are found in the chloroplast and are directly involved in photosynthesis. However, under stress conditions such as high light intensity and nutrient deficiency, the production of primary carotenoids may increase in order to dissipate the excess energy [4]. Moreover, some photosynthetic microorganisms accumulate large amounts of secondary carotenoids in their cells, as a mechanism of photoprotection. In particular, under high light stress conditions, the dissipation of the excess absorbed light energy occurs via the non-photochemical quenching (NPQ) of chlorophyll fluorescence, a harmless nonradiative pathway of energy dissipation. This defensive strategy involves the synthesis of antioxidant carotenoids, such as the secondary carotenoid astaxanthin, lutein and the xanthophyll cycle pigments, i.e., zeaxanthin, violaxanthin and antheraxanthin [4].

Carotenoids extracted from algae are commercially important: β-carotene (IUPAC name β,β-carotene) from *Dunaliella* is used in health foods as a vitamin A precursor, while astaxanthin (IUPAC name (3*S*,3′*S*)-3,3′-dihydroxy-β,β-carotene-4,4′-dione) from *Haematococcus pluvialis* is used in aquaculture for coloring the flesh of fish (mainly salmon). Additionally, astaxanthin—which is the most valuable of the carotenoids—is sold as a nutraceutical; given that it is a powerful antioxidant, it may improve immune system health and protect against neurodegenerative conditions and cancer. Lutein (IUPAC name β,ε-carotene-3,3’-diol) represents another promising target product for algae cultivation. The lutein market is segmented into pharmaceutical, nutraceutical, food, pet foods, and animal and fish feed. In addition to its use as a nutraceutical (lutein may help slow the progression of age-related macular degeneration) and a food coloring agent, newer applications are emerging in cosmetics and as an antioxidant. Currently lutein is produced commercially by extraction from marigold flowers; however, the lutein content of marigolds is low (0.03% dry wt.) which renders alternative lutein sources, such as algae, of interest [5].

Since 2009, we have been investigating the use of microalgae for the beneficial re-use of carbon emissions from point sources such as power plants [6,7,8]. During the late summer/early fall months of algae cultivation at East Bend Station, a coal-fired power plant located in northern Kentucky, USA, a native alga invaded the *Scenedesmus acutus* culture, subsequently identified as *Coelastrella* sp. When stressed, red-orange pigment(s) are produced within the cell. In this context we note that there is recent literature describing the formation of astaxanthin and other carotenoids in *Coelastrella rubescens* [9,10,11], *Coelastrella striolata var. multistriata* [12], *Coelastrella oocystiformis* [13], *Coelastrella* sp. F50 [14] and sp. M60 [15], *Coelastrella* sp. KGU-H001 [16], *Coelastrella terrestris* [17], *Coelastrella astaxanthina* sp. nov. [18], as well as several other *Coelastrella* isolates [19,20,21]. Indeed, it has been suggested that *Coelastrella* could be a viable candidate for commercial production of carotenoids. In addition, the extract from *Coelastrella* sp. F50 has been demonstrated to display antitumor activity in hepatocellular carcinoma [22].

In this contribution, we present data pertaining to the characterization of this *Coelastrella* isolate, in terms of its molecular phylogeny, morphology, pigment content and fatty acid composition. The potential of this novel strain as a source of natural pigments was investigated, focusing on its growth in different cultivation conditions and the impact of stress conditions on pigment accumulation.

## 2. Results and Discussion

### 2.1. Taxonomic Putative Identification

**Figure 1:**
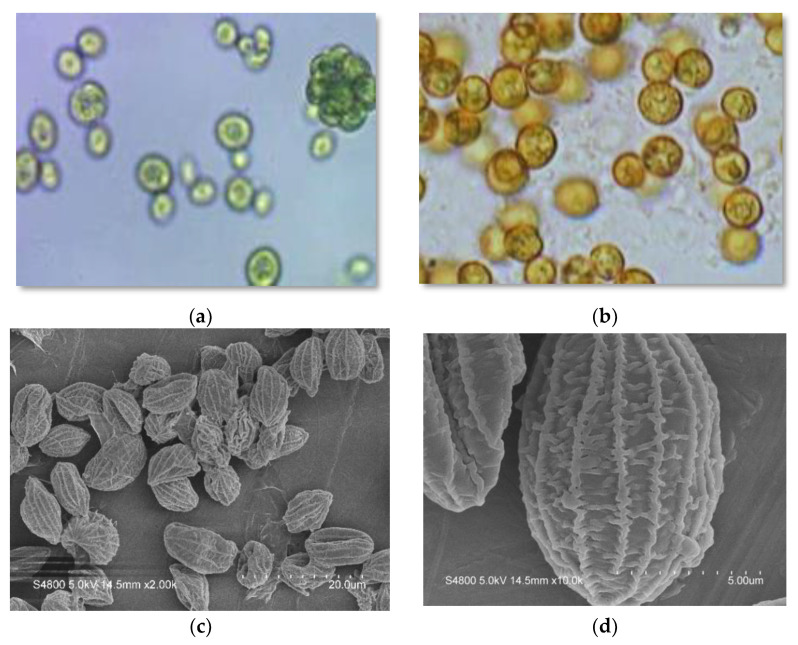
Micrographs of green and red *Coelastrella* cells obtained by optical and scanning electron microscopy: (**a**,**c**,**d**): “green” cells at end of exponential growth phase; (**b**): “red” cells after 12 days of stressing (see Figure 4 for details).

Optical and SEM images of the cells are shown in Figure 1. Cell morphologies ranged from spherical to ellipsoidal, the cells dividing by forming autospores numbering between 4 and 16 (Figure 1a). The surface topology of the cells was visible in SEM images, the cells exhibiting meridional ribs that run from one pole to the other (Figure 1d). These features have been described for other *Coelastrella* species and are evidently typical of the genus [18,20,21,23,24].

The genus *Coelastrella* belongs to the phylum Chlorophyta, to the order Sphaeropleales and the family Scenedesmaceae. Using de novo sequencing, ribosomal and ITS sequences of the isolate were determined (hereafter denoted as contig 1 and contig 2). Both alleles have the best BLAST hits and alignments to *Coelastrella*. Moreover, such results indicate that it is an unknown strain and potentially a unique species. A BLAST and Clustal Omega study of assembled ITS regions of the unknown provided further insight into the identity of this organism. BLAST indicated closest match to Coelastrella, a subsequent multiple alignment using 14 sequences (derived from full length ITS for closely related genus and species) being performed. The resulting maximum likelihood phylogenetic trees for contig 1 and 2 are shown in Figures S1 and S2, the closest relative being *Coelastrella aeroterrestrica* strain Ru-1-8 with 97% identity for the two contigs analyzed, suggesting the classification of Coelastrella sp. for the organism.

### 2.2. Media Screening

**Figure 2:**
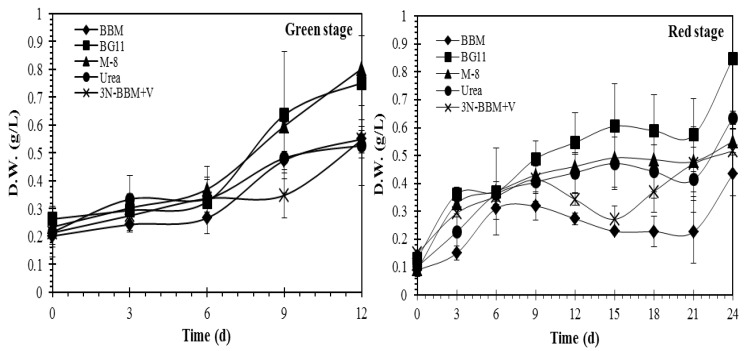
Effect of different media on the growth (dry weight) of *Coelastrella* sp. in the vegetative and carotenogenesis stages. Culturing in the vegetative (“green”) stage used 5 standard media recipes under 40 μmol photons m^−2^ s^−1^ illumination, 16:8 (light:dark) photoperiod, for 12 days. For the carotenogenesis (“red”) stage, cells grown under vegetative conditions were centrifuged, washed of all media, and re-suspended in BG-11 medium without nitrate but with addition of 0.15 M NaCl and subjected to 240 μmol m^−2^ s^−1^ continuous illumination for 24 days. Results represent mean ± SD of three replicates.

Experiments were conducted to compare the effect of growth conditions (nutrient availability) on pigment formation as well as to compare strategies for inducing carotenogenesis by means of stressing. Literature studies indicate that increased salinity and light intensity are key factors in inducing pigment formation, while the depletion of specific nutrients (nitrogen and phosphorus) can also be an important factor [25,26]. As shown in Figure 2, cell growth in the vegetative stage was observed in each of the five media tested, the highest growth being observed in BG-11 and M-8 media according to dry weight (DW) measurements. As expected, growth during the subsequent carotenogenesis stage was significantly lower (due to nitrogen depletion of the cells) although the BG-11 and M-8 media again provided the highest growth.

The effect of the different culture media (BBM, BG-11, M-8, urea and 3N-BBM+V media) on the growth of *Coelastrella* sp. in the vegetative and carotenogenesis stages is summarized in Table 1. In the vegetative stage, biomass productivity of 47.25 ± 3.48 mg L^−1^ d^−1^ and 39.34 ± 2.5 mg L^−1^ d^−1^ was observed for cultivation in M-8 and BG11 media, respectively, with significantly higher yields than for the other media investigated (*p* < 0.05). Cultures grown in BG-11 and urea media in the vegetative stage and then re-suspended in BG-11 medium without nitrate showed the highest biomass productivity (29.82 ± 0.74 mg L^−1^ d^−1^ and 22.22 ± 1.07 mg L^−1^ d^−1^, respectively) in the carotenogenesis stage.

**Figure 3:**
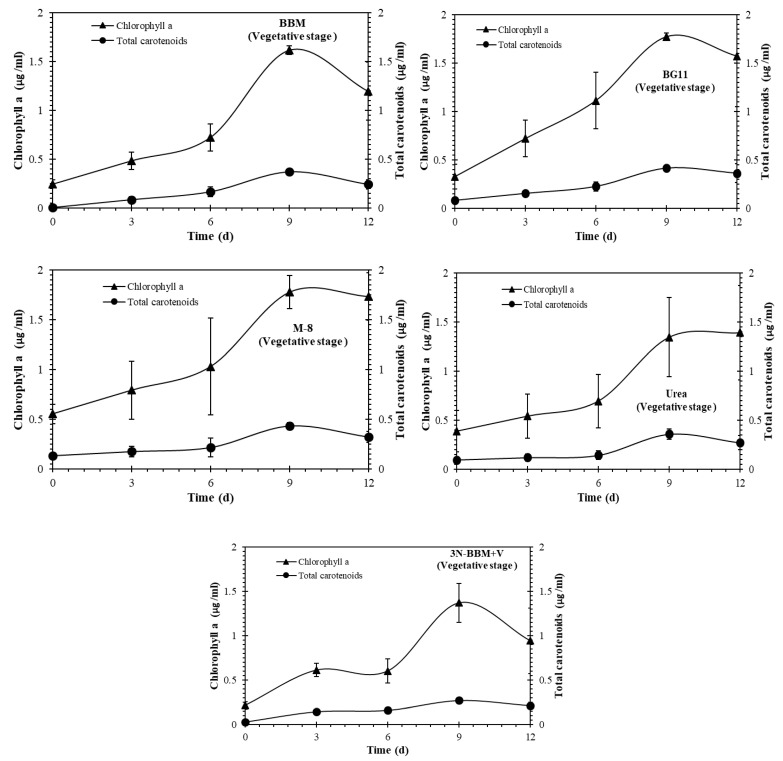
Chlorophyll a and carotenoid production in *Coelastrella* sp. in the vegetative stage during cultivation in different media. The vegetative stage involved culturing under 40 μmol m^−2^ s^−1^ illumination, 16:8 (light:dark) photoperiod, for 12 days. Cultures were maintained in a mixed state by bubbling with a mixture of 3% CO_2_ in N_2_. Results represent mean ± SD of three replicates.

**Figure 4:**
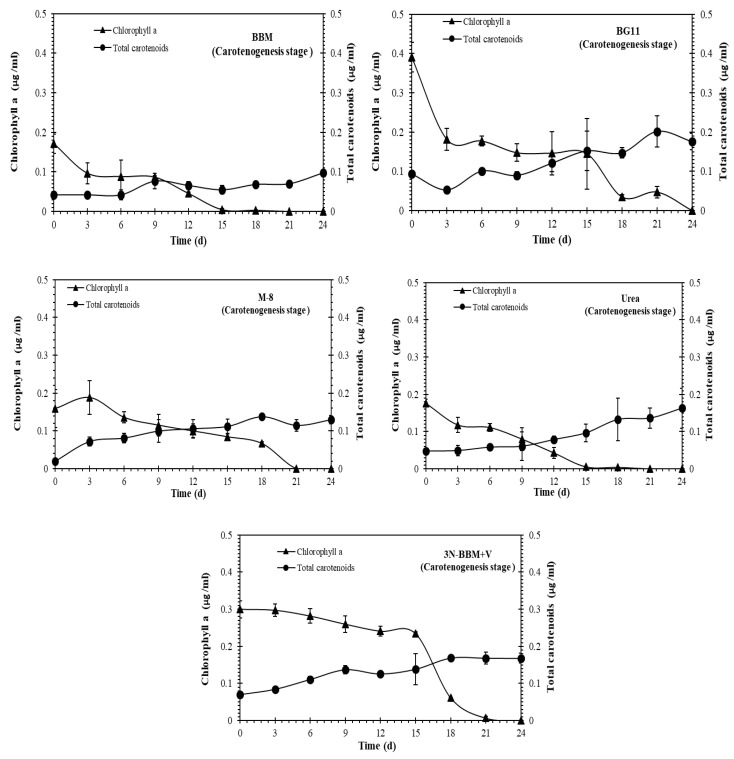
Chlorophyll a and carotenoids production in *Coelastrella* sp. in the carotenogenesis stage. After pre-cultivation in 5 standard medias at 40 μmol m^−2^ s^−1^, 16:8 (light:dark) photoperiod for 12 days, algal cells were transferred and cultured for 24 days on the BG-11 medium without nitrate, with the addition of 0.15 M NaCl and 24 h light at 240 μmol m^−2^ s^−1^. Cultures were maintained in a mixed state by bubbling with a mixture of 3% CO_2_ in N_2_. Results represent mean ± SD of three replicates.

As shown in Figure 3 and Figure 4, chlorophyll a and carotenoids production in the both vegetative and carotenogenesis stages was observed for all of the cultures. The highest total carotenoid concentrations, expressed in µg/mL, were observed in the BG-11 and BBM media in the vegetative stage (Figure 3). During carotenogenesis (Figure 4) total carotenoid concentrations increased in all of the media, while chlorophyll a declined to zero by day 24 in all cases. The highest carotenoid concentrations in this stage were observed for *Coelastrella* sp. cultured in BG-11, 3N-BBM+V and urea media.

### 2.3. Effect of N and P Concentrations on Growth and Effect of Stressing Conditions on Carotenoid Production

**Figure 5:**
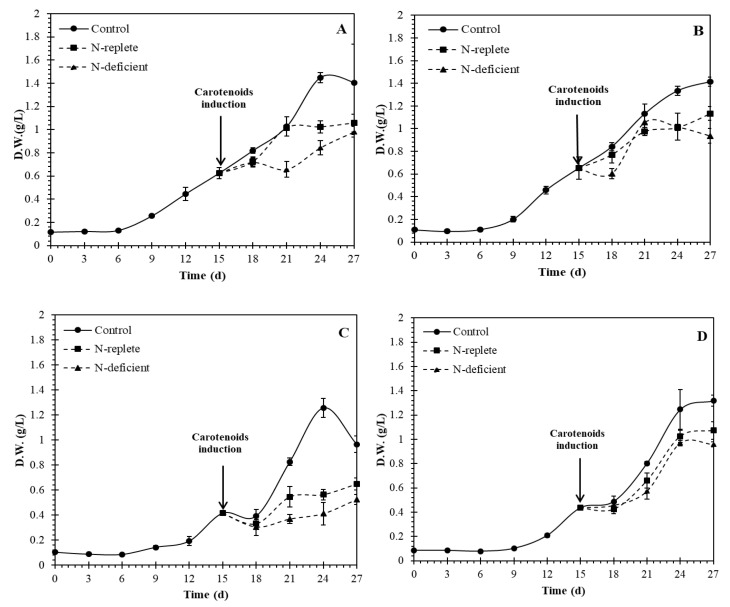
Growth curves (dry weight in g/L) of *Coelastrella* sp. cultured on BG-11 medium modified with different nitrogen and phosphorus concentration combinations (A: N+P-, B: N-P+, C: N-P-, D: N+ P+) for 15 days and then further cultured for 12 days using three different strategies to induce carotenogenesis: control, N-replete and N-deficient. Cultures were maintained in a mixed state by bubbling with a mixture of 3% CO_2_ in N_2_.

To investigate the effects of nitrogen and phosphorus concentrations on growth and carotenoid production in *Coelastrella* sp., the alga was batch cultured in 1 L conical flasks using BG-11 medium modified with four different combinations of nitrogen and phosphorus concentrations, A–D (see Experimental Section). After 15 days, three different cultivation strategies were compared for inducing carotenoid production. In the first, the culture was maintained under the same conditions as a control. In the second strategy (N-replete), culturing was conducted with the addition of 1.5% NaCl and exposure to increased light intensity of 500 μmol m^−2^ s^−1^, while in the third strategy (N-deficient), the culture was harvested, centrifuged, washed of all previous media and re-suspended in the four modified BG-11 media combinations but without nitrogen, with the addition of 1.5% NaCl, and with exposure to increased light intensity of 500 μmol m^−2^ s^−1^ (Figure 5).

The growth curves depicted in Figure 5 show that the control group maintained the highest productivity throughout, followed by the N-replete and the N-deficient groups. The media recipes utilized in this research were all formulated to provide sufficient nutrients to the culture up to a density of 1 g L^−1^, which is made clear by the slowed growth as each group reached 1 g L^−1^. The exception to this was the case of media combination C (N-P-), where the lack of available nitrogen and phosphorus limits the growth to a maximum of approximately 0.5 g L^−1^ in the allotted time. Furthermore, as shown in Table 2, the growth parameters were significantly (*p* < 0.05) affected by the N/P ratio in the medium and by the cultivation strategy. Table 2 confirms that the highest biomass productivity, specific growth rate and biomass yield were obtained under the control conditions, when the algal cells were grown on medium containing a high concentration of nitrogen or phosphorus, or both.

**Figure 6:**
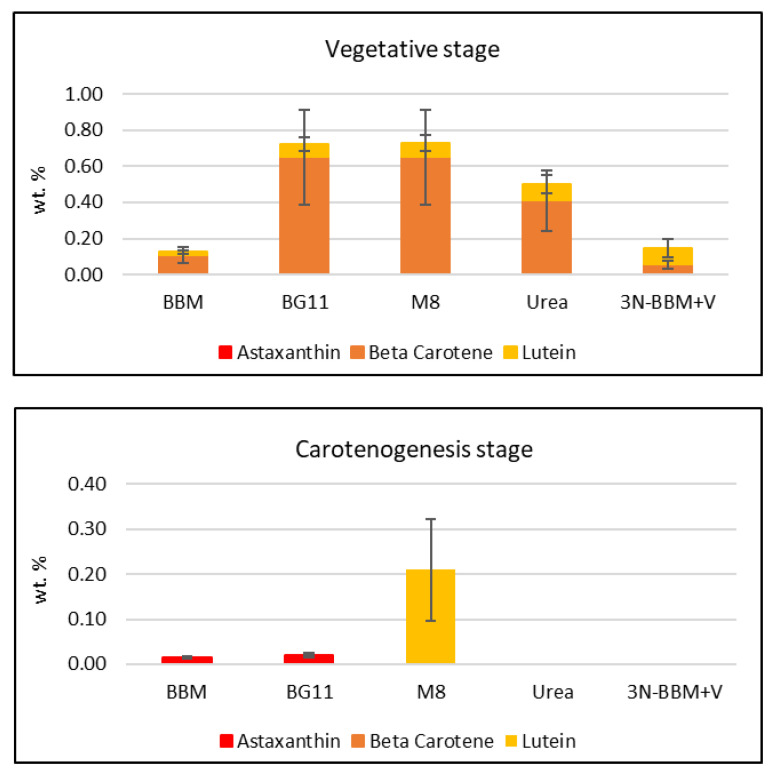
Carotenoids produced by *Coelastrella* sp. in the initial screening of 5 standard media in the vegetative stage (after 12 days) and the carotenogenesis stage (after an additional 24 days). Error bars represent the coefficient of variation.

Detailed analysis of the carotenoids formed in these experiments was performed using HPLC (see Figure S3 for a representative chromatogram). HPLC carotenoid standards selected for the analysis were astaxanthin, β-carotene and lutein, as they currently hold the highest market value [1]. As shown in Figure 6, the total amount of these three carotenoids produced in the vegetative phase of the initial media screening experiments (Section 2.2) was observed to be highest in the groups cultured in BG-11 and M-8 media, broadly following the trend established for total carotenoids by spectrophotometry (Figure 3). Total carotenoids produced in the subsequent carotenogenesis stage of the media screening were much lower overall, with the highest observed being produced in the M-8 medium. Previous studies [26] have shown that primary carotenoids, such as lutein, degrade under stress conditions and therefore their concentration is decreased.

**Figure 7:**
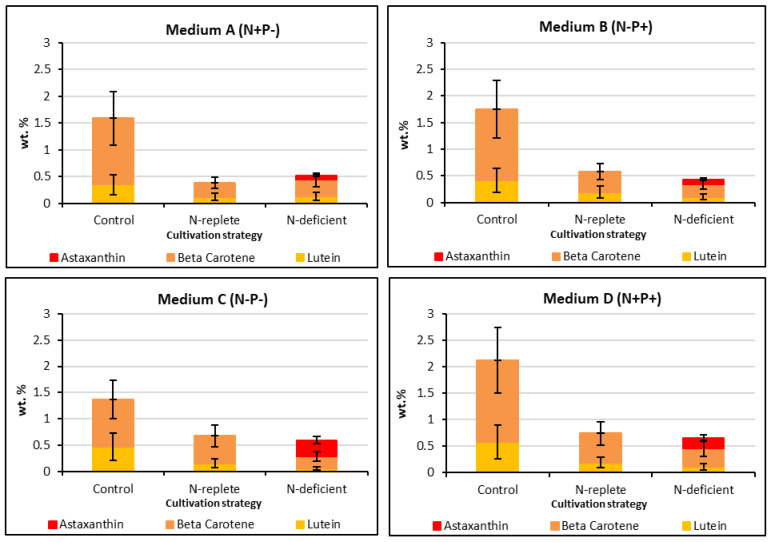
Carotenoids produced by *Coelastrella* sp. from the four BG-11 modified media combinations of N/P concentrations (A: N+P-, B: N-P+, C: N-P-, D: N+ P+) and further sub-categorized by the three subsequent carotenogenesis strategies: control, N-replete, N-deficient. Error bars represent the coefficient of variation.

Figure 7 summarizes the carotenoid concentrations from the four BG-11 modified media combinations of N/P concentrations, the results being further sub-categorized by the three different cultivation strategies used during carotenogenesis: control, N-replete and N-deficient. Considering the control group, it is apparent that the highest β-carotene and lutein concentrations were accumulated under N- and P-replete conditions (Medium D). Conversely, the lowest β-carotene and lutein concentrations were obtained under N- and P-deficient conditions (Medium C). As for the initial media screening experiments (Figure 6), these results suggest that β-carotene and lutein accumulation are favored under conditions that are optimal for cell growth, consistent with their role in photosynthesis [4]. In contrast, the results in Figure 7 clearly show that astaxanthin accumulation in *Coelastrella* sp. requires high stress conditions, and specifically, nitrogen deficiency. Moreover, the requirement for nitrogen deficiency combined with high light intensity and/or NaCl addition is indicated, given that astaxanthin was not produced in the control experiment performed under N-deficient conditions (Medium C). It is also noteworthy that the highest astaxanthin concentration was accumulated when the organism was first cultured under N-deficient conditions in the vegetative phase; this would result in the lowest intracellular N concentrations at the start of the carotenogenesis phase of the experiments depicted in Figure 6, and hence, the most severe nitrogen deficiency condition.

Carotenoid concentrations reported for *Coelastrella* sp. vary widely, although the β-carotene and lutein concentrations reported in Figure 7, corresponding to maximum values of 1.54 and 0.58 wt. % (dry biomass), respectively, fall at the upper end of literature reports and are comparable to those reported for *Coelastrella* sp. F50 [14]. The maximum astaxanthin concentration recorded was 0.31 wt. %; given the value of this product, the use of optimized carotenogenesis conditions, e.g., employing increased salinity and/or light intensity, would be of interest.

According to the literature, the first step of astaxanthin synthesis from β-carotene involves two hydroxylation reactions, this pathway being promoted by the addition of NaCl. *Coelastrella* sp. KGU-Y002 cells cultured with NaCl are reported to accumulate zeaxanthin and lutein, the former being an intermediate in the oxidative processes which lead to the biosynthesis of astaxanthin [27]. Under excess light and nitrogen deficiency stresses, additional reactive oxygen species (ROS) such as hydrogen peroxide are generated by the algae cells. As a defense against these ROS, additional carotenoids are produced along a biochemical pathway mediated by abscisic acid [28], a pathway which is promoted by the presence of additional salt. The resulting increase in abscisic acid concentration triggers an increased counter response to the stresses and thus more carotenoids are generated [29]. Hence, N-deficiency by itself would be not expected to induce astaxanthin formation, as indeed is the case for this *Coleastrella* strain (*vide supra*). It has been observed that phosphorus limitation correlates with slow growth rate and low culture density, whereas abundant nitrogen and phosphorus levels provide optimum growth conditions [30]; however, this does not appear to have a linear effect on the carotenoid production reported in Figure 7.

### 2.4. Lipid Production

**Figure 8:**
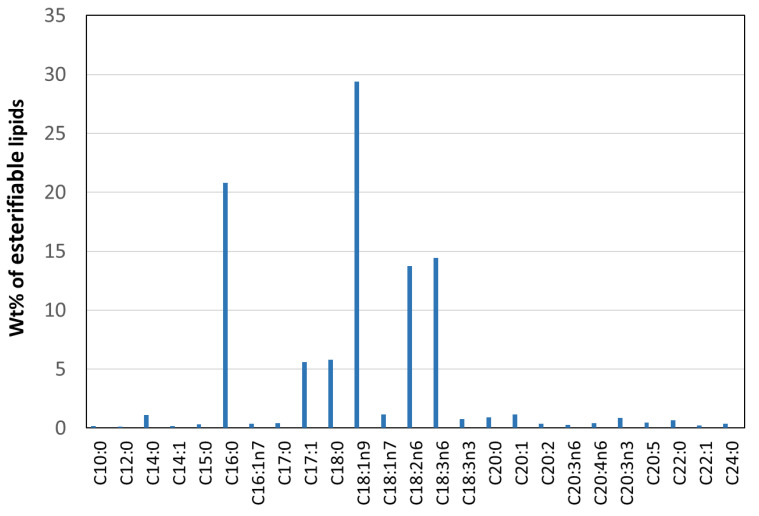
Lipid profile of *Coelastrella* sp.

The composition of *Coelastrella* sp. that was cultivated using urea media (vegetative state) was analyzed and found to correspond to 24.5% total lipids, 14.7% protein, 50.5% carbohydrate and 10.3% other constituents. The esterifiable lipid profile was determined by means of simultaneous in situ transesterification and fatty acid methyl ester (FAME) extraction [31] (Figure 8), esterifiable lipids constituting 17.2 ± 0.5% of the biomass by weight. Analysis of the FAME composition showed the presence of twenty-five identified fatty acid chains, the FAMEs mostly containing saturated (SFAs, ~30.7%) and unsaturated (USFAs, ~69.3%) fatty acid chains with chain lengths from C10 to C24. The predominant component was oleic (C18:1, 29.4 ± 1.3%) followed in descending order by palmitic (C16:0, 20.8 ± 0.8%), γ-linolenic (C18:3, 14.5 ± 0.6%), linoleic (C18:2, 13.7 ± 0.6%), stearic (C18:0, 5.8 ± 0.3%) and margaric (C17:1, 5.6 ± 0.1%). The percentage of other fatty acid chains was relatively low.

## 3. Materials and Methods

### 3.1. Reagents

Five media recipes were chosen based on the literature [32,33,34,35] for biomass productivity optimization experiments (Table 3). Chemicals used in the media were as follows: CaCl_2_.2H_2_O (65%), KOH (85%), FeCl_3_.6H_2_O (97+%), ZnCl_2_ (98+%), FeSO_4_.7H_2_O (99.5%), Na_2_MoO_4_.2H_2_O (99+%), CoCl_2_.6H_2_O (technical grade), and Al_2_(SO_4_)_3_.18H_2_O (“Extra Pure”) were obtained from Acros Organics headquartered in Morris, NJ. H_3_BO_3_ (98%) was obtained from Alfa Aesar headquartered in Ward Hill, MA. Vitamin B12 (Cyanocobalamin) (98+%) and Co(NO_3_)_2_.6H_2_O (98%) were also obtained from Alfa Aesar. CuSO_4_.5H_2_O (98%) was obtained from BDH VWR analytical headquartered in Randor, PA. K_2_HPO_4_.3H_2_O and Fe-EDTA (both technical grade) were obtained from Beantown Chemical headquartered in Hudson, NH. Vitamin B1 (thiamin hydrochloride) was obtained from Calbiochem headquartered in San Diego, CA. MnCl_2_.4H_2_O (97%) was obtained from City Chemical headquartered in West Haven, CT. EDTANa_2_ (95%) was obtained from Fisher Scientific headquartered in Hampton, NH. Urea (99.2%), NaNO_3_ (99%), NaCl (99.7%), KH_2_PO_4_ (99.9%), EDTA (100.9%), MgSO_4_.7H_2_O and ZnSO_4_.7H_2_O were likewise obtained from Fisher Scientific. Ammonium ferric citrate (14.5–16%) was obtained from Honeywell Research Chemicals headquartered in Morris Plains, NJ. Na_2_CO_3_ (99.5%) was obtained from Ricca Chemical Company headquartered in Arlington, TX. Citric acid (99.5%) was obtained from Spectrum Laboratory Products headquartered in New Brunswick, NJ. H_2_SO_4_ (36.5–38%) and Na_2_CO_3_ (99% pure) were obtained from VWR headquartered in Radnor, PA.

Dimethylsulfoxide (99%) was obtained from Alfa Aesar. Acetonitrile (99.5%) and ethyl acetate (99.55) were obtained from Honeywell Research Chemicals headquartered in Morris Plains, NJ. Pigment standards used were astaxanthin (97%) obtained from Adipogen Life Sciences (headquartered in San Diego, CA, USA), β-carotene (97%) obtained from TCI America (headquartered in Portland, OR), and lutein (90%) from Acros Organics. Reagents used in the determination of FAME profiles were as follows: chloroform (HPLC grade, Acros Organics), methanol (HPLC grade, Fisher Chemical), hexane (HPLC grade, Sigma Aldrich), HCl (36.5%–38%, J.T. Baker). GC standards comprised tridecanoic acid methyl ester and FAME Standard Calibration Mix C8:0–C24:0, which were both obtained from Sigma Aldrich.

### 3.2. Molecular Identification

DNA was extracted using MagAttract HMW DNA Kit (Qiagen, Hilden, Germany) following the manufacturer’s user guide. The DNA was eluted in 100 uL AE buffer. The concentration of DNA was evaluated (Table 3) using the Qubit^®^ dsDNA HS Assay Kit (Life Technologies, Carlsbad, USA). The library was prepared using Nextera DNA Flex library preparation kit (Illumina) following the manufacturer’s user guide. An amount of 50 ng DNA was used to prepare the library. The sample underwent the simultaneous fragmentation and addition of adapter sequences. These adapters are utilized during a limited-cycle PCR in which unique index was added to the sample. Following the library preparation, the final concentration of the library (Table 3) was measured using the Qubit^®^ dsDNA HS Assay Kit (Life Technologies), and the average library size (Table 3) was determined using the Agilent 2100 Bioanalyzer (Agilent Technologies, Santa Clara, USA). The library was diluted (to 0.6 nM) and sequenced paired end for 500 cycles using the NovaSeq system (Illumina, San Diego, USA).

To gain some genetic insight into the possible identity of the unknown organism we assembled (NGEN V17 DNAstar) two unique ITS regions from illumina shotgun data. These ITS regions were entered into NCBI BLAST against nr database (July 2020). BLAST data was used to compute a pairwise alignment between a query and the database sequences searched. Note this does not explicitly compute an alignment between the different database sequences (i.e., does not perform a multiple alignment). For the purposes of this sequence tree presentation an implicit alignment between the database sequences is constructed, based upon the alignment of those (database) sequences to the query. Only the higher scoring sequences were included in the trees using a maximum sequence difference of 0.05%. This analysis indicates that the closest genus to the unknown sequences is *Coelastrella* sp.

Subsequently an actual alignment of two uniquely assembled ITS regions assembled from shotgun illumina data was performed using CLustal OMEGA. Alignment of 14 sequences with closest identity and with full sequence lengths were chosen and aligned with the unknown contigs (after manually editing all sequences to the same length (>3000 bp) with 5′ and 3′ ends that had 100% identity). A phylogenetic tree was created using RAxML with 500 bootstraps and random seed. The sequence with the closest relationship to both denovo ITS regions of the unknown was MH703752.1 *Coelastrella aeroterrestrica* strain Ru-1-8.

### 3.3. Optical Microscopy and SEM Analysis

The microalga morphology was analyzed using a microscope (Nikon Labophot-2, Tokyo, Japan) with total magnification of 400×. Photomicrographs were taken with a Nikon Labophot-2 with an AmScope MU1400 digital microscope camera attached to the microscope. Algae samples for SEM analysis were isolated by filtration and re-suspended in 1% glutaraldehyde and left overnight in a refrigerator. The samples were then isolated by filtration and were washed three times with distilled water. Next, the algae were dehydrated by rinsing (on the filter) with a series of EtOH/water mixtures increasing in alcohol concentration (50%, 75%, 90%, 100% *v*/*v*). Each rinsing step lasted about 10 min. Samples were allowed to air dry overnight, after which the constituent cells were coated with Au in a sputter system. Electron micrographs were obtained using a Hitachi S4800 (model) field-emission SEM operated under vacuum. A lower accelerating voltage of 5 kV was employed to prevent algae microstructure damage by the electron beam, resulting in high quality SEM images showing surface ultrastructure.

### 3.4. Protocol for Media Screening Experiments

The microalga *Coelastrella* sp. was cultured at room temperature using a gas mixture containing 3% CO_2_ and 97% N_2_ [32]. Algae cultivation was performed in 800 mL glass columns using Bold’s basal medium (BBM), BG-11, M-8, urea medium, and 3N-BBM+V with each of these media possessing different nitrogen/phosphorus ratios (Table 4) [32,33,34,35,36]. All media were prepared and sterilized in triplicate. For the screening study, 800 mL glass columns were incubated using 40 μmol m^−2^ s^−1^, with a 16:8 h (light: dark) photoperiod for 12 days in 5 standard media. The light was provided by cool-white fluorescent lamps. The growth was monitored every 3 days by means of spectrophotometry using the optical density at 680 nm (OD_680_) and dry weight (DW) (as outlined below). The cells were harvested after 12 days. Harvested cells were centrifuged, washed of all media, and re-suspended in the BG-11 medium without nitrate with addition of 0.15 M NaCl. The cultures were incubated at 25 °C under continuous illumination (240 μmol m^−2^ s^−1^) for 24 days. The growth was again monitored every 3 days by OD_680_ and DW. Pigment analysis was also carried out every 3 days in both the vegetative and carotenogenesis stages. Analysis for carotenoids by HPLC was conducted at the end of the vegetative stage and again at the end of the carotenogenesis stage.

### 3.5. Protocol to Test Effect of Nitrogen and Phosphorus Concentrations on Growth and Stressing Conditions on Carotenoid Production

*Coelastrella* sp. was batch cultured in 1 L conical flasks using BG-11 medium modified with four different combinations of nitrogen and phosphorus concentrations, A-D (Table 5). All flasks were incubated at 40 μmol m^−2^ s^−1^ with 24 h illumination and were mixed by bubbling using a mixture of 3% CO_2_ in N_2_ gas for 15 days. Next, each combination of flasks was divided into three groups: (i) control, in which the culture was maintained under the same conditions; (ii) N-replete, in which culturing was performed with the addition of 1.5% NaCl and exposed to light intensity of 500 μmol m^−2^ s^−1^; and (iii) N-deficient, in which the culture was harvested, centrifuged, washed of all previous media and re-suspended in BG11 medium without nitrogen with addition 1.5% NaCl, and exposed to light intensity of 500 μmol m^−2^ s^−1^. All cultures were maintained in a mixed state by bubbling with a mixture of 3% CO_2_ in N_2_ gas. Monitoring and analysis were conducted as described above.

### 3.6. Growth Measurements

#### 3.6.1. Optical Density (OD)

The culture growth was determined by measurement of OD_680_ nm spectrophotometrically using 3 mL of culture at intervals of 3 days.

#### 3.6.2. Dry Weight (DW)

The DW was measured gravimetrically every 3 days over the period of cultivation; culture samples (20 mL suspensions) were filtered through pre-weighed filter paper (0.45 μm) and washed with deionized water. The filter was oven-dried at 60 °C, cooled, then weighed. The biomass yield (*BY*) (g L^−1^) and productivity (*BP*) (mg L^−1^ d^−1^), specific growth rate (μ, d^−1^) and doubling time (t_d_, d) were calculated according to Equations (1)–(4) [37].

(1) $$BY=\left(X_{f}-X_{0}\right)$$

(2) $$BP=\frac{\left(X_{f}-X_{0}\right)}{\left(T_{2}-T_{1}\right)}$$

*X_f_* and *X*_0_ are the concentrations of biomass (g L^−1^) at the end and beginning of a batch run and *T*_1_ and *T*_2_ represent the incubation period of an experiment where *T_1_* is the initial time (i.e., day 0) and *T*_2_ is the final day of incubation. Specific growth rate (μ, d^−1^) of the microalga was calculated according to the following formula:

(3) $$µ=\frac{1}{t}*ln\frac{x_{f}}{x_{0}}$$

*X_f_* and *X*_0_ are the concentrations of biomass (g L^−1^) at the end and beginning of a batch run, respectively, and t is the duration of the run. Doubling time (t_d_, d) was calculated from:

(4) $$t_{d}=\frac{0.6931}{\mu }$$

where μ is specific growth rate (d^−1^).

### 3.7. Pigment Extraction and Quantification

Pigments were extracted from algal cells using dimethylsulfoxide (DMSO, 99%). Culture samples (5 mL) were centrifuged at 5000 rpm for 5 min and the supernatant discarded. Hot (60 °C) DMSO (5 mL) was added and the cells were re-suspended by vortexing. Samples were then incubated at 60 °C, with occasional shaking, for 10 min before centrifuging again. The supernatant containing the extracted pigment was then decanted [38]. The OD at 649, 665 and 480 nm was determined and the pigment content calculated using the equations below [39]:

(5) $$\mathrm{Chlorophyll}\;a\;\left(\mathrm{Chl}\;a\right)\left(µg\;ml^{-1}\right)=12.47\;\left(OD_{665}\right)-3.62\;\left(OD_{649}\right)$$

(6) $$\mathrm{Chlorophyll}\;b\;\left(\mathrm{Chl}\;b\right)\left(\mu g\;ml^{-1}\right)=25.06\;\left(OC_{649}\right)-6.5\;\left(OD_{665}\right)$$

(7) $$\mathrm{Total}\;\mathrm{carotenoids}\;\left(µg\;ml^{-1}\right)=\frac{\left[1000\;\left(OD_{480}\right)-1.29\;\left(\mathrm{Chl}\;a\right)-53.78\;\left(\mathrm{Chl}\;b\right)\right]}{220}$$

### 3.8. Pigment Analysis by High Performance Liquid Chromatography (HPLC)

Pigment content was also analyzed by HPLC using an Agilent 1260 Infinity Quaternary LC system (Santa Clara, USA) equipped with a G1311A Quaternary pump, G1329B Autosampler, G1364C Fraction Collector, G1316A Column Compartment, and G1315C Diode-Array Detector (DAD) (Agilent system, Santa Clara, USA). The analytical method employed an Agilent ZORBAX Eclipse Plus C18 Reversed-phase column (4.6 mm × 250 mm, 5 μm) and guard column (4.6 mm × 12.5 mm, 5 μm), this being an adaptation of a previously reported method [40]. Freeze-dried algae samples were ground under liquid nitrogen and approximately 10 mg of each sample was added to 500 μL of acetone and deionized H_2_O (9:1) followed by sonication for 15 min. Algal suspensions were then centrifuged in a Thermo Scientific^TM^ Sorvell^TM^ ST 16 centrifuge at 3000 rpm for 5 min, from which the supernatant was decanted into an amber sample vial followed by the addition of fresh acetone solution, and sonication, centrifugation, and decantation as described above. The combined supernatant for each sample was then subjected to HPLC analysis. A solvent mixture of acetonitrile, ddH_2_O, and ethyl acetate was used to elute the pigments. The solvent concentrations and gradients were 90% acetonitrile, 10% ddH_2_O from 0 to 1 min; 86% acetonitrile, 9.6% ddH_2_O, and 5% ethyl acetate from 2 to 14 min; 100% ethyl acetate from 15 to 17 min. A post-run with the initial solvent mixture followed for 3 min. The flow rate was constant at 1.0 mL min^−1^. Pigments were detected at λ = 445 nm with a reference at λ = 550 nm. The concentrations of individual pigments were determined from the HPLC profiles calibrated with standard samples of the individual carotenoids [41]. Note that this analytical method analyses for (all-E)-carotenoids; significant quantities of (Z)-isomers were not detected (see Figure S3). Owing to the fact that in some of the analyses there were fewer than three replicates available, the coefficients of variation for the measured pigment concentrations (wt%) were estimated from those cases where three replicates were obtained. The estimated coefficients of variation for wt% lutein, β-carotene and astaxanthin values were, respectively, 0.54, 0.40 and 0.22 and reflect variability in the efficiency with which the pigments were extracted using the above method. 

### 3.9. Lipid, Protein and Total Carbohydrate Analysis

The lipid profile and total esterifiable lipid content of *Coelastrella* sp. as determined by in situ transesterification [41]. Protein content was determined using Bio-Rad protein reagents according to a published method [42] and total carbohydrates were determined by spectrophotometric quantification as monosaccharides after complexation with 3-methyl-2-benzothiazolinone hydrazone (MBTH) [43].

### 3.10. Statistical Analysis

All the experiments were conducted in triplicate. One-way ANOVA was used to determine the significant difference in dependent variables, and Tukey’s test at a reliability level (of *p* < 0.05) was used to identify differences between each level of treatment. The statistical analyses were performed using Minitab software (V18, Minitab Inc., State College, PA, USA).

## 4. Conclusions

In this work a new *Coelastrella* isolate was studied. Alignment of two uniquely assembled ITS regions of the organism was performed using CLustal OMEGA, the sequence with the closest relationship to both denovo ITS regions of the isolate being *Coelastrella sp.* with high similarity to C. *aeroterrestrica* strain Ru-1-8. Maximum biomass productivity for the alga was achieved in the vegetative stage when culturing was performed in BG-11 or M-8 media. β-carotene and lutein accumulation was favored under conditions that were optimal for cell growth, corresponding to N- and P-replete BG-11 medium. In contrast, astaxanthin accumulation in *Coelastrella* sp. required high stress conditions, and specifically, nitrogen deficiency. Moreover, the requirement for nitrogen deficiency combined with high light intensity and/or NaCl addition is indicated, given that astaxanthin was not produced in a control experiment in which only N-deficiency was applied. Under all conditions, β-carotene and lutein were the major pigments formed, reaching maximum concentrations of 1.54 and 0.58 wt. % (dry biomass), respectively. Additionally, under nitrogen-replete conditions, *Coelastrella* sp. was found to accumulate significant amounts of esterifiable lipids, containing mainly C16-C18 fatty acid chains. Combined with its high growth rate, these characteristics suggest that this *Coelastrella* strain is an interesting candidate for biorefinery applications.

## Figures and Tables

**Table 1:** Kinetics of *Coelastrella* sp. growth in different culture media.

Media *	Growth Stage	Biomass Productivity (mg L^−1^d^−1^)	Specific Growth Rate (µ) (d^−1^)	Doubling Time (t_d_) (d)	Biomass Yield (g L^−1^)
BBM	vegetative stage	29.00 ± 1.01 ^b^	0.083 ± 0.002 ^ab^	8.31 ± 0.17 ^ab^	0.35 ± 0.01 ^b^
BG-11		39.34 ± 2.57 ^a^	0.087± 0.004 ^ab^	7.96 ± 0.41 ^ab^	0.49 ± 0.04 ^a^
M-8		47.25 ± 3.48 ^a^	0.102 ± 0.009 ^a^	6.80 ± 0.69 ^b^	0.57 ± 0.04 ^a^
Urea		25.83 ± 4.58 ^b^	0.074 ± 0.010 ^b^	9.49 ± 1.21 ^a^	0.31 ± 0.06 ^b^
3N-BBM+V		27.72 ± 4.49 ^b^	0.078 ± 0.009 ^b^	8.99 ± 1.80 ^ab^	0.33 ± 0.05 ^b^
					
BBM/BG-11	carotenogenesis stage	14.37 ± 3.42 ^c^	0.066 ± 0.011 ^ab^	10.66 ± 1.96 ^b^	0.36 ± 0.08 ^c^
BG-11/BG-11		29.82 ± 0.74 ^a^	0.077 ± 0.001 ^a^	8.93 ± 0.14 ^b^	0.72 ± 0.02 ^a^
M-8/BG-11		19.13 ± 1.91 ^bc^	0.075 ± 0.003 ^a^	9.26 ± 0.38 ^b^	0.46 ± 0.05 ^bc^
Urea/BG-11		22.22± 1.07 ^b^	0.076 ± 0.004 ^a^	9.09 ± 0.50 ^b^	0.53 ± 0.03 ^b^
3N-BBM+V/ BG-11		15.28 ± 2.97 ^c^	0.051 ± 0.005 ^b^	13.60 ± 1.21 ^a^	0.37 ± 0.07 ^c^

* For the carotenogenesis stage, cells grown under vegetative conditions were centrifuged, washed of all media, and re-suspended in the BG-11 medium without nitrate but with addition of 0.15 M NaCl. Results represent mean ± SD of three replicates. ^abc^ Values for each stage within the same column bearing different superscripts are significantly different (*p* < 0.05).

**Table 2:** Kinetics of *Coelastrella* sp. growth cultivated with the four BG-11 modified media (A:N+P-, B:N-P+, C:N-P-, D:N+P+) and further sub-categorized by the three subsequent carotenogenesis strategies: control, N-replete, N-deficient. Results represent mean ± SD of three replicates.

Media Combinations	Carotenogenesis Strategy	Biomass Productivity (mg L^−1^ d^−1^)	Specific Growth Rate (u) (d^−1^)	Doubling Time (td) (d)	Biomass Yield (g L^−1^)
Medium A	Control	47.78 ± 2.13 ^a^	0.092 ± 0.003 ^a^	7.52 ± 0.25 ^b^	1.29 ± 0.06 ^a^
	N-replete	34.84 ± 2.76 ^b^	0.081 ± 0.003 ^b^	8.50 ± 0.34 ^b^	0.94 ± 0.07 ^b^
	N-deficient	31.97 ± 1.85 ^b^	0.079 ± 0.004 ^b^	8.80 ± 0.41 ^a^	0.86 ± 0.05 ^b^
Medium B	Control	48.33 ± 1.48 ^a^	0.095 ± 0.002 ^a^	7.29 ± 0.16 ^b^	1.31 ± 0.04 ^a^
	N-replete	46.48 ± 2.43 ^a^	0.094 ± 0.003 ^a^	7.39 ± 0.27 ^b^	1.26 ± 0.07 ^a^
	N-deficient	30.68 ± 2.63 ^b^	0.079 ± 0.004 ^b^	8.70 ± 0.47 ^a^	0.82 ± 0.07 ^b^
Medium C	Control	31.97 ± 2.69 ^a^	0.083 ± 0.005 ^a^	8.35 ± 0.53 ^b^	0.86 ± 0.07 ^a^
	N-replete	20.22 ± 1.76 ^b^	0.068 ± 0.004 ^b^	10.14 ± 0.55 ^a^	0.55 ± 0.05 ^b^
	N-deficient	15.59 ± 1.60 ^b^	0.061 ± 0.004 ^b^	11.49 ± 0.83 ^a^	0.42 ± 0.04 ^b^
Medium D	Control	45.64 ± 1.74 ^a^	0.101 ± 0.004 ^a^	6.85 ± 0.29 ^b^	1.23 ± 0.05 ^a^
	N-replete	36.56 ± 2.67 ^b^	0.093 ±0.004 ^ab^	7.42 ± 0.34 ^ab^	0.99 ± 0.07 ^b^
	N-deficient	32.31 ± 0.92 ^b^	0.089 ± 0.004 ^b^	7.76 ± 0.37 ^a^	0.87 ± 0.02 ^b^

^abc^ Values for each strategy within the same column bearing different superscripts are significantly different (*p* < 0.05).

**Table 3:** DNA, final library concentration, and average library size.

DNA Concentration (ng/µL)	Final Library DNA Concentration (ng/µL)	Average Library Size (bp)
1.5	15.00	673

**Table 4:** Composition of media used in vegetative stage cultivation.

Media	NO_3_ (g/L)	Urea (g/L)	PO_4_ (g/L)	N/P Ratio
BBM	0.25	-	0.25	1.0
BG-11	1.5	-	0.04	37.5
M-8	3.0	-	1.0	3.0
Urea	-	0.55	0.12	4.58
3N-BBM+V	0.75	-	0.15	5.05

**Table 5:** Nitrogen and phosphorus concentration combinations used in modified BG-11 cultivation media.

Concentration Combinations	NaNO_3_ (g/L)	K_2_HPO_4_ (g/L)	N/P Ratio
(A) N-replete + P-deficient	1.5	0.04	37.5
(B) N-deficient + P-replete	0.38	0.16	2.38
(C) N-deficient + P-deficient	0.38	0.04	9.5
(D) N-replete + P-replete	1.5	0.16	9.38

## Data Availability

The datasets generated and/or analyzed during the current study are available from the corresponding author on reasonable request.

## Supplementary Materials

The following supporting information can be downloaded at: https://www.mdpi.com/article/10.3390/molecules27206950/s1, Figure S1. Maximum likelihood phylogenetic tree for contig 1 based on a denovo of ribosomal and ITS sequences. Scale bar = 0.01 substitutions per site. Figure S2. Maximum likelihood phylogenetic tree for contig 2 based on a denovo of ribosomal and ITS sequences. Scale bar = 0.1 substitutions per site. Figure S3. HPLC chromatogram of carotenoid extract from Coelastrella sp. subjected to carotenogenesis in N- and P-deficient BG-11.

## Sample Availability

Samples of the compounds are not available from the authors.
