# Supplementary material for: Formation of Lutein, β-Carotene and Astaxanthin in a Coelastrella sp. Isolate
Source: Molecules. 2022 Oct 17;27(20):6950. doi: 10.3390/molecules27206950 (PMC9608941; doi:10.3390/molecules27206950)
Supplement: Supplementary file 1 [file molecules-27-06950-s001.zip › molecules-1941716-Figures S1 and S2.pdf]

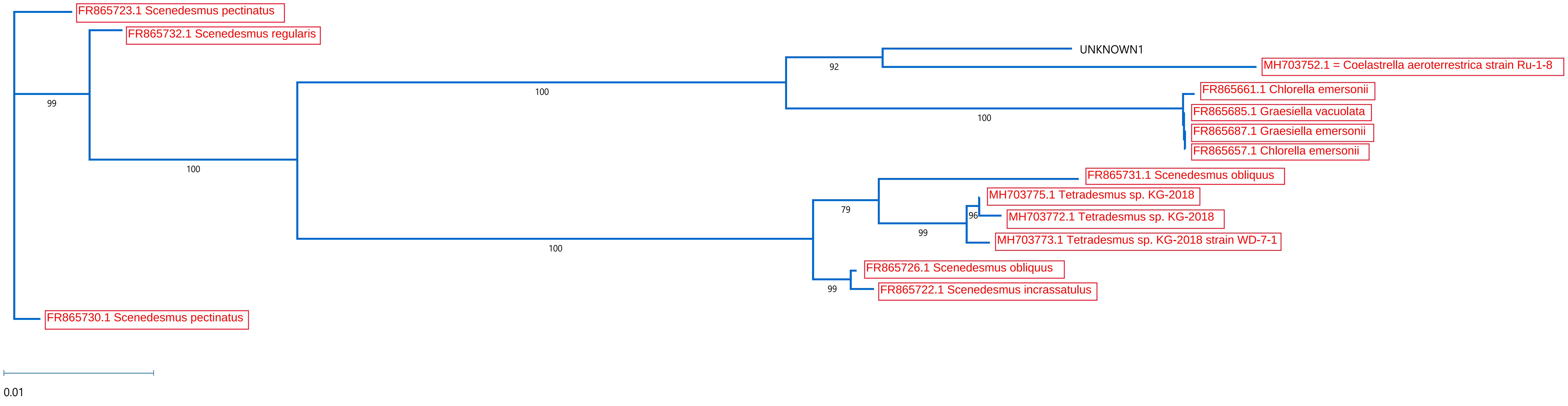

Figure S1. Maximum likelihood phylogenetic tree for contig 1 based on a denovo of ribosomal and ITS sequences. Scale bar = 0.01 substitutions per site.

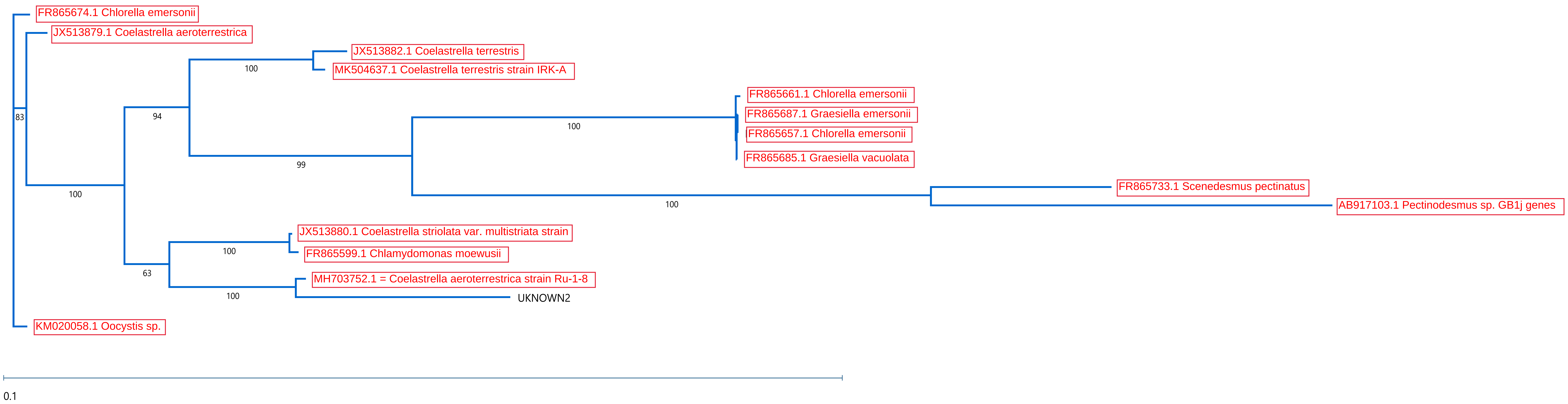

Figure S2. Maximum likelihood phylogenetic tree for contig 2 based on a denovo of ribosomal and ITS sequences. Scale bar = 0.1 substitutions per site.
