# Supplementary figures and images for: Formation of Lutein, β-Carotene and Astaxanthin in a Coelastrella sp. Isolate
Source: Molecules. 2022 Oct 17;27(20):6950. doi: 10.3390/molecules27206950 (PMC9608941; doi:10.3390/molecules27206950)

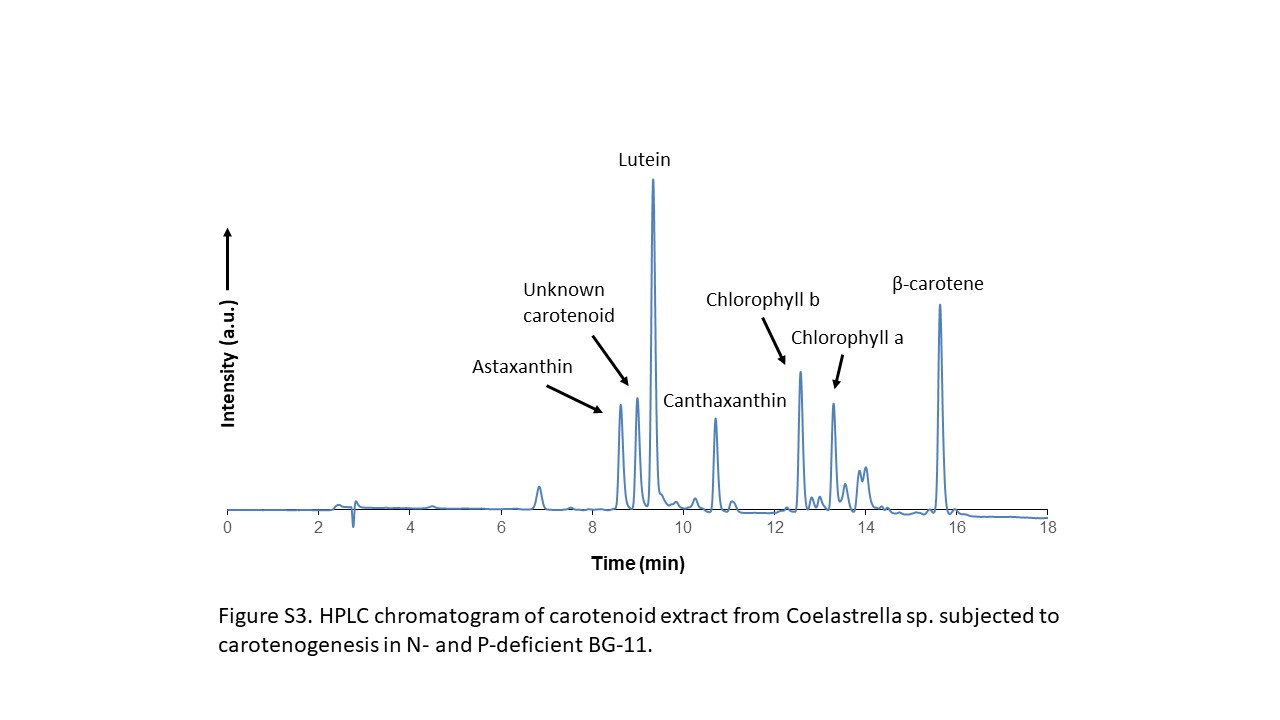

Supplement: Supplementary file 1 [file molecules-27-06950-s001.zip › molecules-1941716-Figure S3.jpg]
